# Pancreatitis in multiple acyl CoA dehydrogenase deficiency: An underdiagnosed complication

**DOI:** 10.1002/jmd2.12175

**Published:** 2020-10-19

**Authors:** Nour Elkhateeb, Anupam Chakrapani, James Davison, Stephanie Grunewald, Spyros Batzios

**Affiliations:** ^1^ Department of Paediatric Metabolic Medicine Great Ormond Street Hospital NHS Trust London UK

**Keywords:** multiple acyl‐CoA dehydrogenase deficiency, MADD, pancreatitis, riboflavin responsiveness, electron transfer flavoproteins

## Abstract

**Background:**

Multiple acyl‐CoA dehydrogenase (MADD) deficiency represents a rare fatty acid oxidation disorder where sporadic reports of pancreatitis already exist. Here, we report three cases of MADD with pancreatic involvement raising questions whether this represents an incidental finding or it is related to the pathophysiology of MADD.

**Methods:**

We have retrospectively studied the clinical, biochemical and radiologic data of patients with MADD diagnosed in our department over the last 20 years to identify patients with pancreatic involvement.

**RESULTS:**

Three out of 17 patients had pancreatic involvement. All three patients were diagnosed with MADD in the neonatal period (two‐third symptomatic—riboflavin nonresponsive, one‐third asymptomatic via newborn screening—riboflavin responsive). Age at presentation of pancreatitis ranged from 20 months to 11 years. Presentations included a single episode of acute pancreatitis in the first patient, chronic necrotizing pancreatitis in the second patient, while the third patient was diagnosed with chronic pancreatitis (CP) incidentally through ultrasonography. All patients had inflammation features on either abdominal computed tomography or ultrasound. Pancreatic enzymes were elevated in two patients. Management of pancreatitis was done conservatively while the patient with necrotic CP required subtotal pancreatectomy.

**DISCUSSION:**

Our data suggest that pancreatitis might be more common in patients with MADD than previously reported, requiring a high index of suspicion in patients with acute metabolic decompensation or nonspecific abdominal symptoms. We hypothesize that the underlying mechanism of pancreatitis in MADD is similar to that in mitochondrial disorders, both resulting from disordered energy metabolism and oxidative phosphorylation.

## 1 BACKGROUND

Multiple acyl‐CoA dehydrogenase deficiency (MADD; MIM #231680) is a rare mitochondrial fatty acid oxidation (FAO) disorder caused by pathogenic mutations in the electron transfer flavoprotein genes (ETFs; ETFA or ETFB) or ETF dehydrogenase (ETFDH).^1^ These flavoproteins are essential for electron transfer from multiple FAD‐linked acyl‐CoA dehydrogenases of β‐oxidation to the respiratory chain.^2^ The disrupted transfer of electrons generated by dehydrogenation reactions, to the mitochondrial respiratory chain leads to impaired mitochondrial FAO and accumulation of short‐, medium‐, and long‐chain acyl‐carnitines in various tissues and lipid accumulation in skeletal muscles, while impaired amino acid and choline metabolism represent additional pathways affected in the disease.^3^ MADD patients show a wide spectrum of clinical severity and variable presentations which are divided based on age of onset in two groups: the neonatal‐onset type with/without congenital anomalies (type I/II) and the late onset subgroup, with manifestations in adolescence or adulthood (type III).^1^ Nonketotic hypoglycemia, metabolic acidosis, hypotonia, hepatic dysfunction, cardiomyopathy, myopathy, exercise intolerance, and rhabdomyolysis represent the most common features of MADD.^2^, ^3^ Patients are additionally categorized according to riboflavin responsiveness.^4^

Pancreatitis is an inflammatory condition of the pancreas with variable involvement of peripancreatic tissues and/or distant organ systems.^5^ It is classified into three categories: acute pancreatitis (AP), acute recurrent pancreatitis (ARP), and chronic pancreatitis (CP).^6^ Etiologies in pediatric patients include gall stones, drug induced pancreatitis, trauma, autoimmune systemic disorders and less commonly, infections, metabolic disorders, and hereditary pancreatitis due to genetic abnormalities.^7^

Despite being an uncommon etiology of pancreatitis, various inborn errors of metabolism (IEMs) have been reported to be associated with it. Lipid disorders, organic acidemias, mitochondrial disorders, glycogen storage disease type 1, Wilson's disease, homocystinuria, and acute intermittent porphyria are the most common IEMs where pancreatic involvement has been reported.^5^, ^6^, ^7^, ^8^, ^9^ Pancreatitis has also been described as one of the rare complications of MADD.^10^, ^11^ Herein, we report three cases of MADD patients with pancreatitis, raising the question whether this represents an incidental finding or is related to the pathophysiology of the disease.

## 2 PATIENTS AND METHODS

We have retrospectively studied the clinical, biochemical and radiological data of patients with MADD diagnosed in our department over the last 20 years to identify patients with pancreatic involvement. Diagnosis of pancreatitis was made based on INSPPIRE criteria.^12^ According to that, diagnosis of AP requires two of the following criteria: (a) characteristic abdominal pain (epigastric or right upper quadrant with/without radiation to the back), (b) serum amylase and/or lipase values three or more times the upper limit of normal (c) diagnostic imaging findings (ultrasound, magnetic resonance imaging, or computed tomography [CT]) compatible with AP. ARP is defined as two or more separate episodes of AP with normalization of serum pancreatic enzyme levels or complete resolution of pain for ≥1 month in between the episodes. CP is characterized by at least one of the following: (a) abdominal pain consistent with pancreatic origin and imaging findings suggestive of chronic pancreatic damage (including pancreatic calcifications, ductal dilatation, fluid collections and pancreatic fibrosis), (b) evidence of exocrine pancreatic insufficiency and suggestive pancreatic imaging findings, and (c) evidence of endocrine pancreatic insufficiency and suggestive pancreatic imaging findings or surgical or pancreatic biopsy specimen demonstrating histopathological features compatible with CP.^12^

## 3 RESULTS

Here, 17 patients were diagnosed with MADD, and 6 patients were male and 11 females. Eleven patients were riboflavin responsive. Then, 3/17 patients (17.64%) had pancreatic involvement based on the above‐mentioned criteria. The epidemiological, clinical, laboratory, and radiological data of those three patients are presented in Table 1.

**TABLE 1:** Clinical, biochemical, and radiological features of patients diagnosed with MADD and pancreatitis

	Case 1	Case 2	Case 3
Sex	Female	Female	Female
Age at diagnosis of MADD	Day 1	Day 5	Day 2
Mode of diagnosis	Symptomatic	NBS	Symptomatic
Carnitine profile at diagnosis	Abnormal/diagnostic	Abnormal/diagnostic	Abnormal/diagnostic
FAO flux studies	Abnormal/consistent with MADD	Abnormal/consistent with MADD	Abnormal/consistent with MADD
Genetics	ETFDH gene: Homozygous c. 1325C > T p.(Ser442Leu)	Not done	ETFDH gene: Heterozygous mutation in exon 4 (c.413 T > G p.L138R) and heterozygous splicing mutation in intron 12 (c.1690 + 2 T > G)
Riboflavin response	Nonresponsive	Responsive	Nonresponsive
Age at diagnosis of pancreatitis	1.8 y	13 y	4 y
Initial symptoms on presentation of pancreatitis	Acute metabolic decompensation, multiorgan dysfunction	Incidentally discovered on abdominal US	Acute abdomen, feeding intolerance, vomiting, diarrhea
Diagnostic criteria of pancreatitis	Acute pancreatitis: Abdominal pain Raised serum lipase Suggestive Imaging findings	Chronic pancreatitis Abdominal pain Suggestive imaging findings	Chronic pancreatitis with flares of acute pancreatitis: Abdominal pain Raised serum lipase Suggestive imaging findings
Time interval between onset of symptoms and diagnosis	4 d	6 mo	3 mo
Amylase level at presentation (U/L)	52	N/A	223
Lipase level at presentation (IU/L)	391	N/A	1570
Imaging findings	U/S diffuse subcutaneous edema and widespread ascites	U/S the visualized pancreas is diffusely heterogeneous in echotexture suggestive of chronic pancreatitis	CT abdomen: pancreatitis with multiple inflammatory collections within the abdomen and a pancreatic pseudocyst
Treatment	Conservative	N/A	Subtotal pancreatectomy
Duration of longest admission due to pancreatitis	6 mo	N/A	20 mo
IV fluids and TPN management (during pancreatitis episodes)	TPN PN 80 mL/kg Glucose 11 g/kg Lipid 1.5 g/kg Protein 1.1 g/kg Calories: 699 kcal/d Weight: 11.44 kg	N/A	First episode: TPN PN: 60 mL/kg (80% maintenance) Glucose 11.3 g/kg Protein 0.9 g/kg (25% of energy) Lipid 1.2 g/kg (24% of energy) Calories: 841 kcal Weight: 17.8 kg Second episode PN: 70 mL/kg Glucose 11.4 g/kg Protein 1 g/kg Lipid 1.8 g/kg Calories 63.7 kcal Weight: 20.6
Dietary management (during pancreatitis episodes)	Moderate protein and fat restricted diet was reintroduced 725 kcal (63.4 kcal/kg/d) Protein 12.66 g (1.1 g/kg/d) CHO 129 g (11.3 g/kg/d) Fat 17.1 g per day (1.5 g/kg/d)	Unrestricted diet and emergency regime (glucose polymer 25%)	In all episodes: moderate protein and fat restricted diet was reintroduced. Example of diet in the first episode: 1033 kcal (58 kcal/kg/d), protein 15.3 g (0.86 g/kg/d) (25% of energy) CHO 51% of energy Fat 28 g per day (1.57 g/kg/d) (24% of energy)
Duration of TPN dependence	1 mo	N/A	3 mo (first episode) 17 mo (second episode)
Concurrent MADD treatment (during pancreatitis episodes)	Carnitine 50 mg/kg/d Hydroxybutyrate 150 mg/kg/d Ubiquinone 30 mg three times daily	Carnitine 50 mg/kg/d Riboflavin 100 mg twice daily	Carnitine 20 mg/kg/d Hydroxybutyrate 75 mg/kg/d Ubiquinone 30 mg three times daily
Other medical management	N/A	N/A	Octreotide infusion (first episode): 4 mcg/kg/h
Surgical management	N/A	N/A	Partial pancreatectomy
Subsequent course	No further episodes	Chronic pancreatitis	Chronic necrotic pancreatitis

Abbreviations: CT, computed tomography; FAO, fatty acid oxidation; MADD, multiple acyl‐CoA dehydrogenase.

### 3.1 Case reports

#### 3.1.1 Case 1

This is a 4‐year‐old female, born to consanguineous Pakistani parents. Neonatal hypoglycemia in the presence of a right multicystic dysplastic kidney and abnormal results in newborn screening (elevated C8 acylcarnitine with normal C8/10 ratio) raised the suspicion of MADD. The diagnosis was confirmed with a repeat acylcarnitine profile, abnormal FAO flux studies, and genetic analysis. Initial riboflavin administration was discontinued when patient was found to be nonresponsive. She was additionally treated with a fat and protein restricted diet and ketone bodies and ubiquinone. Medium chain triglyceride supplementation was as well used as patient has shown on multiple occasions essential fatty acid deficiency. She was noted to have hypotonia with motor delay and polyneuropathy. No metabolic decompensations were noted till the age of 1.8 years. At this age, she presented with a 4‐day history of vomiting, acute epigastric pain, and feeding intolerance. She was initially managed with intravenous fluids (dextrose 10%, GIR 5.8 mg/kg/min), which was increased to 12.5% (GIR 7.3 mg/kg/min) due to hypoglycemia. She quickly deteriorated with acute metabolic decompensation and multi‐organ dysfunction requiring PICU admission and mechanical ventilation. She required CVVH for fluid management and was started on TPN on which her blood glucose stabilized. Lipase level was 391 IU/L, while amylase was 52 U/L (normal reference range: lipase 10‐150 IU/L, amylase 30‐100 U/L). Abdominal sonography showed diffuse subcutaneous edema and ascites with unremarkable pancreatic appearances; however, further imaging was not performed. Her lipase peaked to 572 IU/L and she finally improved on conservative treatment. Feeds were reintroduced gradually. No further episodes of pancreatitis were documented to date. She remains otherwise stable, with intermittent episodes of hypoglycemia associated with intercurrent illnesses.

#### 3.1.2 Case 2

**FIGURE 1:**
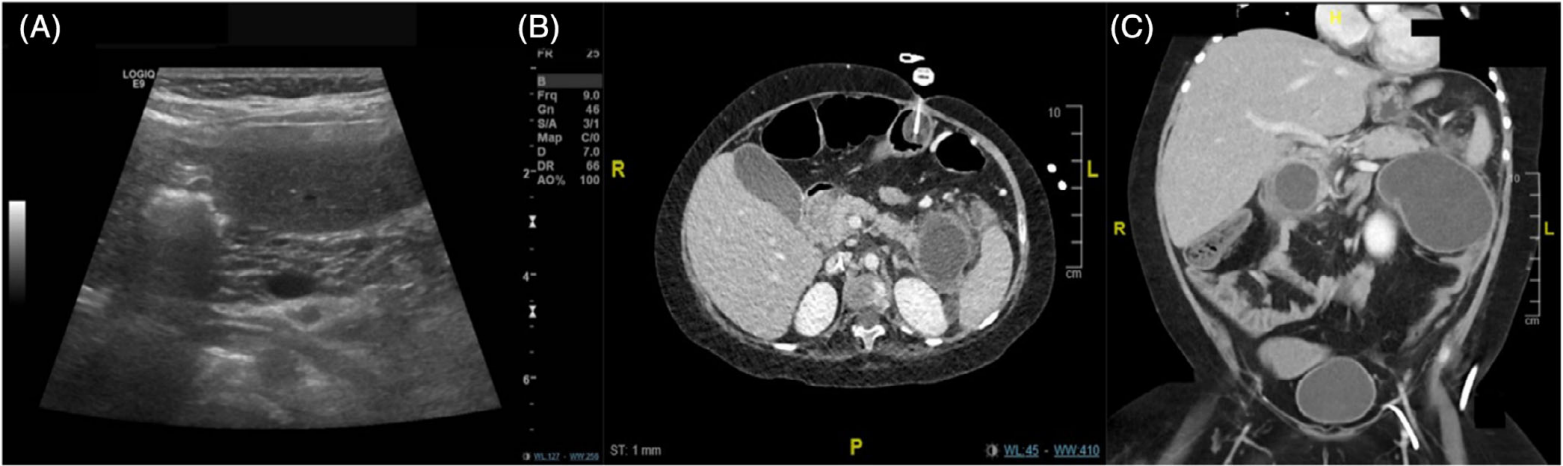
Pancreatic imaging findings. A, Case 2: abdominal ultrasonography: The visualized pancreas is diffusely heterogenous in echotexture with multiple foci of parenchymal low echogenicity in keeping with chronic pancreatitis. B,C Case 3: Contrast enhanced CT abdomen: evidence of an inflammatory thick‐walled pseudocyst in the region of the uncinate process of the pancreas (34 × 34 mm). In addition, there is dilatation and ectasia of the pancreatic duct and a large pseudocyst demonstrated in the pancreatic tail. This extends out to Gerota's fascia on the left side and has a more linear course adjacent to the descending colon to terminate in a very large pseudocyst (measuring 71 × 75 × 87 mm) which extends toward the anterior abdominal wall and reaches almost into the left side of the pelvis. Small inflammatory pseudocysts in the region of the splenic hilum and greater curve of the stomach are seen

This is a 15‐year‐old female born to non‐consanguineous parents of Eritrean origin. She was diagnosed with riboflavin‐responsive MADD after abnormal neonatal screening. Biochemistry and FAO flux studies confirmed the diagnosis. She otherwise had developmental delay and hypermobility attributed to coexistence of Trisomy 21. She responded well to riboflavin supplementation and remained stable during the course of the disease on an unrestricted diet. At the age of 13 years, she was incidentally found to have CP based on ultrasonographic findings showing diffusely heterogeneous pancreas in echotexture with multiple foci of parenchymal and low echogenicity associated with moderate hepatic steatosis (Figure 1A). This was preceded by a 6‐month history of recurrent episodes of abdominal pain, associated with vomiting, that required multiple visits to the emergency department. She additionally had an episode of metabolic decompensation 4 months earlier with elevated CK, transaminitis, and muscle pain, where no definite cause was identified. These episodes were managed by oral emergency regime (glucose polymer 25%). Amylase and lipase were not assessed unfortunately in any of those episodes. To date the patient remains stable with no additional episodes of metabolic decompensation or pancreatic deterioration.

#### 3.1.3 Case 3

This is a 10‐year‐old female that presented on day 2 of life with hypoglycemia and cardiac arrest. Metabolic workup was indicative of MADD and diagnosis was genetically confirmed. She was initially treated with riboflavin but showed no responsiveness. She received a fat and protein restricted diet and ubiquinone. She had quite severe symptoms with pronounced hypotonia and during the course of her disease, she developed hypoventilation due to myopathy, hypothyroidism, osteoporosis, renal tubulopathy and left ventricular hypertrophy. At the age of 4 years, she presented with abdominal pain, vomiting, diarrhea, and feeding intolerance, following a port‐a‐cath infection. Pancreatic enzymes showed a lipase of 1570 U/L and amylase of 223 U/L. Abdominal sonography at this time was normal, yet the pancreas was not visualized. She required TPN with multiple failed trials of feeding reintroduction. Feeds were finally introduced again after 3 months. She received octreotide infusion during this episode for treatment of pancreatitis. At the age of 5 years, she had another episode of AP following an RSV infection. She deteriorated rapidly and developed respiratory distress, metabolic acidosis, elevated CK, and transaminitis. TPN was started with multiple failed trials of feeding reintroduction. CT abdomen showed multiple inflammatory collections, and a pancreatic pseudocyst within the uncinate (Figure 1B,C). Multiple ultrasonographic attempts of drainage of the pseudocyst and intrabdominal collections failed. She underwent a partial pancreatectomy which has not relieved her symptoms. She remained hospitalized for a total of 20 months and finally had to undergo ERCP for stenting of the pancreatic duct, splenectomy, and subtotal pancreatectomy. Since then, no further episodes of pancreatitis have been reported. She suffers from postpancreatectomy diabetes and pancreatic exocrine insufficiency and is on pancreatic enzyme replacement therapy in addition to her regular treatment.

## 4 DISCUSSION

We describe three patients with MADD who developed pancreatitis during the course of their disease. As this accounts for 18% of our MADD patients (3/17), it raises significant questions in regards to the etiopathological mechanisms behind this high incidence of pancreatic involvement. All three patients are females and were diagnosed with MADD as neonates. Age at presentation of pancreatic symptoms ranged from 20 months to 11 years. Early manifestations included abdominal pain, vomiting, and diarrhea. Presentations included a single episode of AP, necrotic CP with recurrent flares of AP, and CP as an incidental finding. Imaging was suggestive of pancreatitis. Serum lipase was elevated in both symptomatic patients, while amylase was increased only in one patient. Time interval between development of pancreatic symptoms and the diagnosis ranged between 4 days and 6 months. The delayed diagnosis of pancreatitis in two patients highlights the importance of having a high index of suspicion in patients with acute metabolic decompensation, or nonspecific abdominal symptoms, to avoid the potential life‐threatening risk associated with missing the diagnosis. Management of pancreatitis followed NICE guidelines^13^ while the patient with necrotic CP finally required partial pancreatectomy. Annular pancreas which is associated with trisomy 21 as a cause of recurrent pancreatitis was excluded in the second patient by abdominal U/S, even though no MRCP was done which would be the imaging modality of choice to exclude this. Other metabolic derangements such as hyperlipidemia, hypertriglyceridemia, hypercalcemia, and diabetes mellitus, which could possibly be the cause of pancreatitis, have been as well excluded in our patients. In addition, none of our patients received any kind of medication reported to increase the possibility of having pancreatitis. A panel of genes associated with hereditary forms of pancreatitis was done in the third patient of our cohort and results were normal.

On review of previously reported cases, pancreatitis associated with MADD was first described in a 2‐year‐old girl presenting with nonketotic hypoglycemia, metabolic acidosis, and finally coma following a respiratory tract infection. Unfortunately, the patient had not survived the episode and pancreatitis was diagnosed as an autopsy finding.^10^ The second report of MADD associated pancreatitis is of a 22‐year‐old female patient, experiencing exercise intolerance since early childhood. She developed her first episode of AP at the age of 11 years and had five additional episodes with complete recovery in between. Abdominal ultrasound revealed enlarged pancreas with calcifications. Diagnosis of MADD was made by organic acid analysis in urine and patient showed a favorable response to riboflavin.^11^ In both cases, diagnosis of pancreatitis preceded that of MADD. On the contrary, in our cohort, MADD diagnosis was established in the neonatal period. There has been a substantial delay in diagnosing pancreatitis in our patients, partially explained by its nonspecific early manifestations. It is also noted that, the manifestations and course of pancreatitis was severe in our patients with riboflavin nonresponsive MADD, with an earlier onset of pancreatitis in childhood, in comparison to the milder course in the responsive patient, with a later onset in adolescence. This is in agreement with the report by Liang et al.^11^

Apart from MADD, pancreatitis has been described in various IBMs, especially in patients with energy metabolism disorders. Among those, mitochondrial disease patients have occasionally pancreatic involvement, with or without exocrine pancreas insufficiency.^9^, ^14^ In some of those patients, pancreatitis manifests with asymptomatic isolated amylase or lipase elevation without imaging abnormalities.^9^ This is similar to our first case, where abdominal ultrasonography failed to reveal findings indicative of pancreatitis, requiring further imaging with abdominal CT.

The pathophysiology of pancreatitis in mitochondrial disorders is presumed to be multifactorial. Acinar cells responsible for the synthesis and secretion of digestive enzymes contain a high density of mitochondria, as they require mitochondrial ATP production to provide sufficient energy for exocytosis of pancreatic digestive proenzymes.^15^, ^16^ In addition, exocytosis requires large cytosolic Ca2+ signals for which energy is provided by mitochondrial ATP production.^15^ It is speculated that pancreatitis in mitochondrial disorders is associated with a metabolic defect in exocrine pancreas cells leading to defective enzyme secretion, finally resulting in intracellular protease accumulation and activation.^9^ In addition, mitochondrial dysfunction and oxidative stress lead to caspase 9 and 3 activation^15^, ^17^ and subsequent activation of other downstream enzymes causing apoptosis or necrosis.^15^, ^18^ Those processes are known to be implicated in the pathogenesis of pancreatic inflammation.^19^ Several studies have suggested that reactive oxygen species (ROS) are involved in the pathogenesis of pancreatitis.^15^, ^20^, ^21^ Respiratory chain enzyme deficiencies increase ROS production, generating superoxide anions and other free radicals. Such oxidative stress has been shown to decrease mitochondrial membrane potential leading to mitochondrial dysfunction and inducing mtDNA damages in acinar cells.^22^ Finally, altered composition of the pancreatic secretions found in mitochondrial disease patients can lead to obstruction of the exocrine pathways and consequently to pancreatitis.^9^

ETF is located in the mitochondrial matrix and receives electrons from several dehydrogenases involved in FAO, choline and amino acid metabolism. Those electrons are transferred to ETFDH, located in the inner mitochondrial membrane, and subsequently, to ubiquinone in the respiratory chain, leading to ATP production.^23^, ^24^ As a result, in MADD, the acyl‐CoA dehydrogenases are unable to transfer electrons generated by dehydrogenation reactions, resulting in the accumulation of various intramitochondrial acyl‐CoA esters.^3^ It is already known that zebrafish mutant in ETFDH and fibroblast cells from MADD patients demonstrate mitochondrial dysfunction, reduced oxidative phosphorylation, increased oxidative stress and dysregulated ROS production.^25^, ^26^, ^27^, ^28^ In addition, upregulation of mitochondrial chaperones indicative of mitochondrial stress was detected in fibroblast studies with downregulation and impaired activity of alpha and beta subunits of ATP synthase complex. Several apoptotic proteins were also found to be overexpressed in MADD fibroblast cells.^28^ As all the above‐mentioned findings have been reported in literature to be involved in the pathogenesis of pancreatitis in mitochondrial disease patients, one can hypothesize that the pathophysiology behind the development of pancreatitis in MADD could have a similar basis. Even patients with riboflavin responsive MADD are known to have chronic persistent mild oxidative stress despite riboflavin treatment,^27^, ^29^ which could possibly explain the occurrence of pancreatitis in riboflavin responsive MADD patients both in our cohort and in literature.

Mitochondrial dysfunction has also been described in other IEMs such as organic acidemias, glycogen storage disease type Ia and hypercholesterolemia with increased mitochondrial oxidative stress, impaired mitophagy, abnormal oxidative phosphorylation, and ultrastructural mitochondrial abnormalities implicated in pathogenesis of secondary mitochondrial dysfunction in these disorders.^30^, ^31^, ^32^, ^33^, ^34^ As pancreatitis was also reported in these disorders, one can hypothesize that there could be a similar pathophysiology behind the development of pancreatitis in various IEMs including MADD.

## 5 CONCLUSIONS

Based on the above‐mentioned findings pancreatitis might be more common in patients with MADD than previously reported, requiring a high index of suspicion in patients with acute metabolic decompensation, or nonspecific abdominal symptoms. This should prompt further assessment with serum amylase/lipase levels and imaging studies. We hypothesize that the pathophysiology and underlying mechanisms of pancreatitis in MADD is similar to that in mitochondrial disorders, both resulting from disordered energy metabolism and oxidative phosphorylation. Further reports are required to study the characteristics of pancreatitis in this rare metabolic condition and confirm the hypothesis that the increased incidence of pancreatitis in MADD is not just coincidental.

## CONFLICT OF INTEREST

The authors declare no conflict of interest.

## AUTHOR CONTRIBUTIONS

**Nour Elkhateeb**: Collected data and wrote the paper. **Anupam Chakrapani**: Reviewed the paper critically and commented for improvement. **James Davison**: Reviewed the paper critically and commented for improvement. **Stephanie Grunewald**: Reviewed the paper critically and commented for improvement. **Spyros Batzios**: Had the original idea for the paper, had the overall supervision, and wrote part of the paper.

## PATIENT CONSENT STATEMENT

Consent was approved from all patients. This article does not contain any studies with human or animal subjects performed by any of the authors.

## ETHICS STATEMENT

Documentation of approval from the Institutional Committee for Care and Use of Laboratory Animals (or comparable committee).

## References

[1] van Rijt WJ , Ferdinandusse S , Giannopoulos P , et al. Prediction of disease severity in multiple acyl‐CoA dehydrogenase deficiency: a retrospective and laboratory cohort study. J Inherit Metab Dis. 2019;42:878‐889. 31268564 10.1002/jimd.12147

[2] Morris AM , Spiekerkoetter U . Disorders of mitochondrial fatty acid oxidation & riboflavin metabolism In: Saudubray JM, Baumgartner MR, Walter J, eds. Inborn Metabolic Diseases Diagnosis and Treatment. Heidelberg: 6th ed, Springer; 2016:201‐214.

[3] Valle D , Beaudet AL , Vogelstein B , et al. Defects of electron transfer flavoprotein and electron transfer flavoprotein‐ubiquinone oxidoreductase: glutaric acidemia type II In: Valle D et al., eds. The Metabolic and Molecular Basis of Inherited Disease. New York: McGraw‐Hill; 2014:2357‐2365.

[4] Horvath R . Update on clinical aspects and treatment of selected vitamin‐responsive disorders II (riboflavin and CoQ 10). J Inherit Metab Dis. 2012;35:679‐687. 22231380 10.1007/s10545-011-9434-1

[5] Majbar AA , Cusick E , Johnson P . Incidence and clinical associations of childhood acute pancreatitis. Pediatrics. 2016;138:e20161198. 27535145 10.1542/peds.2016-1198

[6] Morinville VD , Lowe ME , Ahuja M , et al. Design and implementation of INSPPIRE. J Pediatr Gastroenterol Nutr. 2014;59:360‐364. 24824361 10.1097/MPG.0000000000000417 PMC4141003

[7] Shukla‐Udawatta M , Madani S , Kamat D . An update on pediatric pancreatitis. Pediatr Ann. 2017;46:e207‐e211. 28489228 10.3928/19382359-20170420-01

[8] Kota SK , Krishna SV , Lakhtakia S , et al. Metabolic pancreatitis: etiopathogenesis and management. Indian J Endocrinol Metab. 2013;17:799‐805. 24083160 10.4103/2230-8210.117208 PMC3784862

[9] Finsterer J , Frank M . Gastrointestinal manifestations of mitochondrial disorders: a systematic review. Therap Adv Gastroenterol. 2017;10:142‐154. 10.1177/1756283X16666806 PMC5330602 28286566

[10] Coskun T , Gogus S , Akcoren Z , et al. Acute pancreatitis in a patient with glutaric aciduria type II. Turkish J Pediatr. 1997;39:379‐385. 9339118

[11] Liang WC , Tsai KB , Lai CL , Chen LH , Jong YJ . Riboflavin‐responsive glutaric aciduria type II with recurrent pancreatitis. Pediatr Neurol. 2004;31:218‐221. 15351024 10.1016/j.pediatrneurol.2004.02.015

[12] Morinville VD , Husain SZ , Bai H , et al. Definitions of pediatric pancreatitis and survey of present clinical practices. J Pediatr Gastroenterol Nutr. 2012;55:261‐265. 22357117 10.1097/MPG.0b013e31824f1516 PMC3626452

[13] National Institute for Health and Care Excellence . Pancreatitis (NICE Quality Standard No. 104). 2018 https://www.nice.org.uk/guidance/ng104 28481484

[14] Ishiyama A , Komaki H , Saito T , et al. Unusual exocrine complication of pancreatitis in mitochondrial disease. Brain Dev. 2013;35:654‐659. 23182449 10.1016/j.braindev.2012.10.015

[15] Gerasimenko OV , Gerasimenko JV . Mitochondrial function and malfunction in the pathophysiology of pancreatitis. Pflugers Arch. 2012;464:89‐99. 22653502 10.1007/s00424-012-1117-8

[16] Bolender RP . Stereological analysis of the Guinea pig pancreas. I. Analytical model and quantitative description of non stimulated pancreatic exocrine cells. J Cell Biol. 1974;61:269‐287. 4363955 10.1083/jcb.61.2.269 PMC2109295

[17] Gerasimenko JV , Gerasimenko OV , Palejwala A , Tepikin AV , Petersen O , Watson AJM . Menadione‐induced apoptosis: roles of cytosolic Ca(2+) elevations and the mitochondrial permeability transition pore. J Cell Sci. 2002;115:485‐497. 11861756 10.1242/jcs.115.3.485

[18] Hajnóczky G , Csordás G , Das S , et al. Mitochondrial calcium signalling and cell death: approaches for assessing the role of mitochondrial Ca2+ uptake in apoptosis. Cell Calcium. 2006;40:553‐560. 17074387 10.1016/j.ceca.2006.08.016 PMC2692319

[19] Leung PS , Chan YC . Role of oxidative stress in pancreatic inflammation. Antioxid Redox Signal. 2009;11:135‐165. 18837654 10.1089/ars.2008.2109

[20] Debray FG , Drouin E , Herzog D , et al. Recurrent pancreatitis in mitochondrial cytopathy. Am J Med Genet A. 2006;140:2330‐2335. 17022070 10.1002/ajmg.a.31457

[21] Tsai K , Wang SS , Chen TS , et al. Oxidative stress: an important phenomena with pathogenic significance in the progression of acute pancreatitis. Gut. 1998;42:850‐855. 9691925 10.1136/gut.42.6.850 PMC1727136

[22] Ehlers RA , Hernandez A , Bloemendal LS , Ethridge RT , Farrow B , Evers BM . Mitochondrial DNA damage and altered membrane potential in pancreatic acinar cells induced by reactive oxygen species. Surgery. 1999;126:148‐155. 10455877

[23] Ruzicka FJ , Beinert H . A new iron‐sulfur flavoprotein of the respiratory chain. A component of the fatty acid beta oxidation pathway. J Biol Chem. 1997;252:8440‐8445. 925004

[24] McKean MC , Beckmann JD , Frerman FE . Subunit structure of electron transfer flavoprotein. J Biol Chem. 1983;258:1866‐1870. 6822538

[25] Kim SH , Scott SA , Bennett MJ , et al. Multi‐organ abnormalities and mTORC1 activation in zebrafish model of multiple acyl‐CoA dehydrogenase deficiency. PLoS Genet. 2013;9(6):e1003563. 23785301 10.1371/journal.pgen.1003563 PMC3681725

[26] Song Y , Selak MA , Watson CT , et al. Mechanisms underlying metabolic and neural defects in zebrafish and human multiple acyl‐CoA dehydrogenase deficiency (MADD). PLoS One. 2009;4:e8329. 20020044 10.1371/journal.pone.0008329 PMC2791221

[27] Cornelius N , Corydon TJ , Gregersen N , Olsen RKJ . Cellular consequences of oxidative stress in riboflavin responsive multiple acyl‐CoA dehydrogenation deficiency patient fibroblasts. Hum Mol Genet. 2014;23(16):4285‐4301. 24698980 10.1093/hmg/ddu146

[28] Rocha H , Ferreira R , Carvalho J , et al. Characterization of mitochondrial proteome in a severe case of ETF‐QO deficiency. J Proteomics. 2011;75:221‐228. 21596162 10.1016/j.jprot.2011.04.025

[29] Olsen RK , Cornelius N , Gregersen N . Genetic and cellular modifiers of oxidative stress: what can we learn from fatty acid oxidation defects? Mol Genet Metab. 2013;110:S31‐S39. 24206932 10.1016/j.ymgme.2013.10.007

[30] Stepien KM , Heaton R , Rankin S , et al. Evidence of oxidative stress and secondary mitochondrial dysfunction in metabolic and non‐metabolic disorders. J Clin Med. 2017;6(7):71. 10.3390/jcm6070071 PMC5532579 28753922

[31] Luciani A , Schumann A , Berquez M , et al. Impaired mitophagy links mitochondrial disease to epithelial stress in methylmalonyl‐CoA mutase deficiency. Nat Commun. 2020;11(1):970. 32080200 10.1038/s41467-020-14729-8 PMC7033137

[32] Schwab MA , Sauer SW , Okun JG , et al. Secondary mitochondrial dysfunction in propionic aciduria: a pathogenic role for endogenous mitochondrial toxins. Biochem J. 2006;398(1):107‐112. 16686602 10.1042/BJ20060221 PMC1525008

[33] Farah BL , Sinha RA , Wu Y , et al. Hepatic mitochondrial dysfunction is a feature of glycogen storage disease type Ia (GSDIa). Sci Rep. 2017;7:44408. 28317891 10.1038/srep44408 PMC5357851

[34] McCommis KS , McGee AM , Laughlin MH , Bowles DK , Baines CP . Hypercholesterolemia increases mitochondrial oxidative stress and enhances the MPT response in the porcine myocardium: beneficial effects of chronic exercise. Am J Physiol Regul Integr Comp Physiol. 2011;301(5):1250‐1258. 10.1152/ajpregu.00841.2010 PMC3213933 21865543

